# Supplementary material for: Quality of routine health facility data for monitoring maternal, newborn and child health indicators: A desk review of DHIS2 data in Lumbini Province, Nepal
Source: PLoS One. 2024 Apr 1;19(4):e0298101. doi: 10.1371/journal.pone.0298101 (PMC10984527; doi:10.1371/journal.pone.0298101)
Supplement: S1 Table — (DOCX) [file pone.0298101.s001.docx]

S1 Table: Outliers analysis of maternal health indicators

| District/ month | **Jul/Aug 2021** | **Aug/Sep 2021** | **Sep/Oct 2021** | **Oct/Nov 2021** | **Nov/Dec 2021** | **Dec 2021/Jan 202** | **Jan/Feb 2022** | **Feb/Mar 2022** | **Mar.Apr 2022** | **Apr/May 2022** | **May/Jun 2022** | **Jun/Jul 2022** |
| --- | --- | --- | --- | --- | --- | --- | --- | --- | --- | --- | --- | --- |
| **Four ANC visits as per protocol** | | | | | | | | | | | | |
| Rukum East | 55 | 63 | 53 | 47 | 61 | 52 | 43 | 68 | 55 | 60 | 58 | 65 |
| Rolpa | 304 | 287 | 221 | 237 | 236 | 278 | 237 | 266 | 245 | 256 | 330 | **360** |
| Pyuthan | 285 | 321 | 235 | 217 | 212 | 299 | 261 | 245 | 227 | 219 | 256 | 307 |
| Gulmi | 334 | 311 | 313 | 287 | 267 | 281 | 255 | 265 | 194 | 213 | 252 | 304 |
| Arghakhanchi | 195 | 188 | 191 | 168 | 162 | 197 | 186 | 175 | 133 | 134 | 195 | **242** |
| Palpa | 397 | 417 | 317 | 315 | 293 | 329 | 322 | 304 | 304 | 320 | 376 | **449** |
| Nawalparasi West | 468 | 547 | 433 | 449 | 403 | 514 | 488 | 400 | 388 | **311** | 389 | 451 |
| Rupandehi | 1781 | 1979 | 1784 | 1777 | 1964 | 1884 | 2011 | 1945 | 1599 | 1540 | 1848 | 2073 |
| Kapilbastu | 738 | 894 | 709 | 660 | 800 | 935 | 853 | 802 | 722 | 619 | 738 | 820 |
| Dang | 733 | 678 | 635 | 680 | 622 | 726 | 639 | 615 | 568 | 568 | 672 | **786** |
| Banke | 924 | 1005 | 912 | 842 | 715 | 806 | 880 | 787 | 634 | 657 | 810 | 791 |
| Bardiya | 531 | 523 | 492 | 530 | 503 | 524 | 468 | 462 | **283** | 345 | 437 | 499 |
| **Institutional Deliveries Total** | | | | | | | | | | | | |
| Rukum East | **98** | 64 | 62 | 56 | 59 | 64 | 49 | 51 | 59 | 60 | 52 | 41 |
| Rolpa | **461** | 355 | 283 | 243 | 258 | 259 | 258 | 253 | 256 | 223 | 241 | 214 |
| Pyuthan | **474** | 379 | 360 | 289 | 267 | 303 | 347 | 287 | 284 | 271 | 296 | 313 |
| Gulmi | 282 | 250 | 250 | 234 | 223 | 202 | 221 | 216 | 188 | 151 | 165 | 164 |
| Arghakhanchi | 158 | 151 | 135 | 115 | 116 | 102 | 123 | 123 | 103 | 83 | 112 | 115 |
| Palpa | **504** | 420 | 426 | 401 | 380 | 404 | 324 | 328 | 320 | 303 | 322 | 394 |
| Nawalparasi West | 335 | 383 | 339 | 314 | 284 | 272 | 264 | 265 | 205 | 176 | 209 | 199 |
| Rupandehi | 2632 | 2954 | 3022 | 2724 | 2611 | 2457 | 2417 | 2295 | 2034 | 1795 | 1873 | 2044 |
| Kapilbastu | 832 | **997** | 887 | 768 | 714 | 718 | 817 | 806 | 702 | 562 | 560 | 579 |
| Dang | 709 | 661 | 633 | 590 | 665 | 693 | 655 | 747 | 619 | 629 | 668 | 760 |
| Banke | 2091 | 2091 | 2141 | 2048 | 1740 | 1622 | 1752 | 1576 | 1375 | 1203 | 1291 | 1460 |
| Bardiya | 561 | 450 | 520 | 455 | 386 | 425 | 415 | 405 | 325 | 193 | 273 | 256 |
| **Women received delivery incentive on transportation** | | | | | | | | | | | | |
| Rukum East | **98** | 64 | 62 | 56 | 59 | 64 | 49 | 51 | 59 | 60 | 52 | 41 |
| Rolpa | **461** | 355 | 283 | 242 | 255 | 251 | 257 | 253 | 256 | 223 | 239 | 216 |
| Pyuthan | **474** | 379 | 360 | 289 | 271 | 299 | 347 | 287 | 284 | 271 | 296 | 313 |
| Gulmi | 281 | 255 | 250 | 229 | 224 | 206 | 223 | 218 | 191 | 153 | 167 | 161 |
| Arghakhanchi | 158 | 151 | 135 | 115 | 116 | 102 | 123 | 123 | 103 | 83 | 112 | 115 |
| Palpa | **126** | 107 | 106 | 71 | 68 | 94 | 89 | 72 | 66 | 61 | 78 | 72 |
| Nawalparasi West | 340 | 386 | 341 | 314 | 284 | 272 | 265 | 265 | 187 | 158 | 178 | 187 |
| Rupandehi | 2430 | 2667 | 2745 | 2492 | 2392 | 2227 | 2209 | 2083 | 1878 | 1679 | 1722 | 1857 |
| Kapilbastu | 783 | 958 | 858 | 751 | 687 | 687 | 776 | 723 | 663 | 512 | 491 | 480 |
| Dang | 649 | 587 | 591 | 560 | 631 | 646 | 614 | 715 | 617 | 629 | 657 | **752** |
| Banke | 2065 | 2025 | 2075 | 1980 | 1700 | 1615 | 1685 | 1565 | 958 | 1223 | 1265 | 1288 |
| Bardiya | 569 | 455 | 535 | 460 | 393 | 425 | 417 | 408 | 333 | 197 | 280 | 264 |
| **3 PNC Visits as per Protocol** | | | | | | | | | | | | |
| Rukum East | **95** | 59 | 49 | 48 | 61 | 54 | 64 | 55 | 45 | 50 | 63 | 46 |
| Rolpa | **450** | 385 | 281 | 246 | 261 | 269 | 249 | 295 | 268 | 202 | 244 | 211 |
| Pyuthan | **350** | 293 | 278 | 220 | 198 | 213 | 232 | 226 | 216 | 184 | 214 | 172 |
| Gulmi | 329 | 289 | 287 | 270 | 239 | 218 | 247 | 223 | 219 | 165 | 204 | 168 |
| Arghakhanchi | 185 | 184 | 181 | 144 | 120 | 117 | 165 | 176 | 129 | 124 | 161 | 155 |
| Palpa | **297** | 222 | 238 | 219 | 217 | 247 | 209 | 217 | 206 | 156 | 170 | 157 |
| Nawalparasi West | 258 | 293 | 266 | 240 | 274 | 259 | 321 | 303 | 277 | 221 | 236 | **185** |
| Rupandehi | **662** | 1041 | 1031 | 1030 | 983 | 1112 | 1123 | 1225 | 1079 | 932 | 1034 | 1067 |
| Kapilbastu | 226 | 357 | 411 | 290 | 364 | 448 | 518 | 512 | 415 | 280 | 322 | 414 |
| Dang | 494 | **578** | 470 | 460 | 456 | 500 | 486 | 538 | 461 | 486 | 521 | 538 |
| Banke | 728 | 721 | 746 | 754 | 682 | 568 | 667 | 730 | 531 | 462 | 518 | 550 |
| Bardiya | 353 | 272 | 279 | 264 | 263 | 359 | 417 | **470** | 388 | 247 | 261 | 287 |

*Monthly values in bold indicate a moderate outlier between ±2-3SD from the mean*
