# Supplementary material for: Quality of routine health facility data for monitoring maternal, newborn and child health indicators: A desk review of DHIS2 data in Lumbini Province, Nepal
Source: PLoS One. 2024 Apr 1;19(4):e0298101. doi: 10.1371/journal.pone.0298101 (PMC10984527; doi:10.1371/journal.pone.0298101)
Supplement: S2 Table — (DOCX) [file pone.0298101.s002.docx]

S2 Table: Outlier analysis of child health indicators

| **District / month** | **Jul/Aug 2021** | **Aug/Sep 2021** | **Sep/Oct 2021** | **Oct/Nov 2021** | **Nov/Dec 2021** | **Dec 2021/Jan 202** | **Jan/Feb 2022** | **Feb/Mar 2022** | **Mar/Apr 2022** | **Apr/May 2022** | **May/Jun 2022** | **Jun/Jul 2022** |
| --- | --- | --- | --- | --- | --- | --- | --- | --- | --- | --- | --- | --- |
| **Children Immunized with Measles/Rubella-2^nd^** | | | | | | | | | | | | |
| Rukum East | 95 | 65 | 106 | **11** | **165** | 111 | 94 | 110 | 91 | 74 | 85 | 72 |
| Rolpa | 326 | 343 | 398 | 432 | **582** | 475 | 355 | 377 | 400 | 394 | 409 | 358 |
| Pyuthan | 289 | 306 | 324 | 474 | 510 | 419 | 500 | 407 | 459 | 404 | 345 | 369 |
| Gulmi | 362 | 292 | 326 | 443 | 435 | 423 | 395 | 366 | 395 | 320 | 317 | 310 |
| Arghakhanchi | 168 | 207 | 255 | 255 | 341 | 315 | 266 | 254 | 302 | 236 | 232 | 195 |
| Palpa | 268 | 256 | 284 | 338 | 382 | 404 | 369 | 380 | 341 | 323 | 283 | 227 |
| Nawalparasi West | 353 | 321 | 447 | 520 | 659 | 686 | 619 | 623 | 697 | 613 | 459 | 427 |
| Rupandehi | 1369 | 1017 | 1408 | 1500 | 2106 | 2056 | 1807 | 1921 | 1937 | 1676 | 1523 | 1305 |
| Kapilbastu | 941 | 731 | 927 | 938 | 1318 | 1275 | 1050 | 1232 | 1351 | 1384 | 1317 | 1641 |
| Dang | 890 | 729 | 837 | 709 | **1447** | 1150 | 1042 | 1121 | 1199 | 1035 | 911 | 786 |
| Banke | 594 | 617 | 719 | 520 | 1222 | 1266 | 988 | 1012 | 1050 | 1120 | 893 | 848 |
| Bardiya | 475 | **382** | 546 | 625 | 771 | 803 | 715 | 780 | 811 | 724 | 680 | 513 |
| **Diraahoea cases (2-59 months children) treated with ORS and Zinc** | | | | | | | | | | | | |
| Rukum East | 146 | 121 | 86 | 48 | 74 | 90 | 74 | 136 | 210 | **325** | 188 | 156 |
| Rolpa | 381 | 451 | 296 | 206 | 241 | 259 | 209 | 333 | 587 | 757 | 607 | 634 |
| Pyuthan | 315 | 342 | 232 | 144 | 178 | 241 | 187 | 208 | 421 | 453 | 537 | 501 |
| Gulmi | 267 | 207 | 234 | 185 | 167 | 207 | **128** | 207 | 286 | 257 | 267 | 275 |
| Arghakhanchi | 153 | 84 | 103 | 107 | 105 | 91 | 70 | 126 | 146 | 163 | 177 | 165 |
| Palpa | 169 | 167 | 160 | 102 | 138 | 103 | 91 | 188 | 292 | 251 | 262 | 232 |
| Nawalparasi West | 252 | 197 | 151 | 151 | 192 | 178 | 168 | 199 | 289 | **333** | 249 | 209 |
| Rupandehi | 523 | 601 | 432 | 397 | 505 | 515 | 353 | 460 | 480 | 683 | 618 | 552 |
| Kapilbastu | 906 | 867 | 735 | 680 | 702 | 606 | 489 | 569 | 682 | **1141** | 757 | 610 |
| Dang | 325 | 296 | 292 | 224 | 286 | 273 | 210 | 298 | 527 | 608 | 514 | 491 |
| Banke | 489 | 571 | 398 | 362 | 429 | 504 | 307 | 342 | 556 | 661 | 574 | 456 |
| Bardiya | 333 | 288 | 261 | 159 | 247 | 247 | 189 | 266 | 479 | **643** | 420 | 335 |
| **New Growth Monitoring visit (0-11 Months children)** | | | | | | | | | | | | |
| Rukum East | 116 | 139 | 71 | 51 | 117 | 94 | 84 | 80 | 87 | 89 | 125 | 86 |
| Rolpa | 487 | **547** | 498 | 423 | 454 | 428 | 386 | 440 | 365 | 346 | 352 | 382 |
| Pyuthan | 459 | **554** | 468 | 372 | 406 | 390 | 373 | 422 | 337 | 349 | 384 | 408 |
| Gulmi | 527 | 488 | 498 | 476 | 448 | 417 | 418 | 379 | 393 | 325 | 330 | 360 |
| Arghakhanchi | 353 | **407** | 387 | 268 | 317 | 264 | 273 | 300 | 266 | 258 | 294 | 227 |
| Palpa | 355 | 345 | 370 | 329 | 322 | 242 | 261 | 282 | 280 | 230 | 214 | 210 |
| Nawalparasi West | 478 | 575 | **704** | 625 | 649 | 583 | 503 | 516 | 518 | 440 | 445 | 445 |
| Rupandehi | 2091 | 2283 | 2399 | 2254 | 2483 | 2246 | 1766 | 2079 | 1985 | 1642 | 1668 | 1983 |
| Kapilbastu | 1241 | 1234 | **1399** | 1166 | 1157 | 1115 | 925 | 1068 | 1005 | 935 | 940 | 876 |
| Dang | 1001 | 1357 | 1492 | 1151 | **1668** | 1431 | 1280 | 1284 | 1130 | 999 | 981 | 1080 |
| Banke | 1254 | **1515** | 1061 | 970 | 1263 | 1186 | 786 | 816 | 915 | 724 | 708 | 757 |
| Bardiya | 743 | 622 | 852 | 682 | 956 | 680 | 530 | 773 | 823 | 685 | 503 | 494 |
| **Exclusive Breast-Feeding practice** | | | | | | | | | | | | |
| Rukum East | 86 | 68 | 77 | 76 | 110 | 87 | 45 | 89 | 104 | 58 | 82 | 84 |
| Rolpa | 277 | 229 | 270 | 260 | 256 | 228 | 234 | 279 | 291 | 260 | 250 | 212 |
| Pyuthan | 375 | 275 | 394 | 323 | 328 | 357 | 322 | 360 | 348 | 294 | 303 | 286 |
| Gulmi | 379 | 315 | 300 | 289 | 377 | 380 | 347 | 363 | 437 | 377 | 399 | 349 |
| Arghakhanchi | 269 | 281 | 309 | 264 | 291 | 261 | 244 | **322** | 259 | 259 | 270 | 237 |
| Palpa | 209 | 173 | 183 | 170 | 209 | 172 | 201 | 217 | 193 | 190 | 181 | 178 |
| Nawalparasi West | **637** | 401 | 516 | 349 | 353 | 344 | 317 | 433 | 430 | 435 | 346 | 439 |
| Rupandehi | 996 | 1025 | 992 | 999 | 867 | 825 | 982 | 1068 | 877 | 841 | 993 | 995 |
| Kapilbastu | **871** | 814 | 669 | 522 | 620 | 625 | 601 | 624 | 612 | 677 | 668 | 767 |
| Dang | 390 | 433 | 497 | 713 | 821 | 839 | 623 | 745 | 661 | 660 | 671 | 516 |
| Banke | 770 | 778 | 580 | 369 | 710 | 554 | 478 | 530 | 528 | 584 | 604 | 579 |
| Bardiya | 419 | 389 | 349 | 306 | 270 | 238 | 220 | 384 | 373 | 405 | 343 | 353 |

*Monthly values in bold indicate a moderate outlier between ±2-3SD from the mean*
