# Supplementary material for: Quality of routine health facility data for monitoring maternal, newborn and child health indicators: A desk review of DHIS2 data in Lumbini Province, Nepal
Source: PLoS One. 2024 Apr 1;19(4):e0298101. doi: 10.1371/journal.pone.0298101 (PMC10984527; doi:10.1371/journal.pone.0298101)
Supplement: Attachment — Submitted filename: Response to Reviewers.docx [file pone.0298101.s005.docx]

July 9, 2023

Editor-In-Chief

PLOS ONE Journal

**Subject: Submitting Revised Manuscript for Possible Publication**

# It is known that we have submitted a manuscript entitled “Quality of Routine Health Information for Monitoring Maternal, Newborn and Child Health Indicators: An Analysis of DHIS2 Data in Lumbini Province, Nepal*”* for possible publication on PLOS Journal and review comments has sent to us. Based on this, we have revised our manuscript by incorporating all the given comments. In addition, we have attached the point-by-point responses and explanations here under.

Thank you,

The Authors

**Authors’ responses to review comments:**

First of all, the authors are grateful to the editors and reviewers for taking their time and providing these

interesting and constructive comments that help us improve the quality of our manuscript.

With this, we have tried to address all the comments point-by-point in the revised manuscript as follows.

**Responses to comments by Reviewer #1:**

1- In the abstract, please use the full form of MNCH.

**Response:** By accepting the comment, we mentioned the full form of MNCH in the revised manuscript.

2- In the abstract, results and conclusion, it is not what MNCH datasets and indicators were and how they were compared. What were the data items? Which data were not consistent?

**Response:** Thank you for this comment. We assessed the completeness and timeliness of facility reporting (district wise) considering datasets as listed in DHIS2 (listed datasets relating to MNCH: Safe motherhood, Immunization, Nutrition and CBIMNCI). For the consistency, we selected key MNCH data items (as listed in Table 1). Based on the comment, we slightly modified the manuscript.

3- Please choose appropriate keywords based on the MeSH terms.

**Response:** Thank you for the suggestion. We revised the keywords and used appropriate keywords based on MeSH terms.

4- In the introduction section, there is no adequate information about data quality and related indicators. Moreover, studies related to evaluating data quality of health information systems need to be reviewed and added to the introduction section.

**Response:** This comment is well taken. Information about data quality and studies related to data quality assessments are further reviewed and added to the introduction section.

5- In the methods section, please provide a reference for lines 126-128.

**Response:** This is well taken, and references added for the lines 126-128.

6- The authors need to explain more about Table 1. For me, it is not still clear how the data elements and indicators have been selected.

**Response:** Thank you for this comment. Logic for selecting the data items listed in Table 1 are now clarified in the revised manuscript. Authors now mentioned: The selection of data items takes into account the MNCH continuum of care approach, their ability to cross-verify and measure data quality issues, as well as their importance in program monitoring and evaluation, as stated in national and provincial policy documents.

7- In Table 2, did the authors look for data elements completeness or just the completeness of reporting on a monthly basis? This question is about timeliness and consistency, too.

**Response:** We thank the reviewer for this valid comment. In Table 2, the authors assessed the timeliness and completeness of facility reporting of the MNCH-related datasets on a monthly basis. For consistency, the authors enrolled the data items (Table 1) and assessed the consistency over time, outliers in the current year, consistency between related data and external consistency of data. The Authors explicitly explained the measurement approaches in the revised manuscript.

8- About the consistency of the mean values, the authors need to explain why it was important for them.

**Response:** The importance of assessing consistency of the mean values is now well explained in the revised manuscript. The authors explicitly explained: the consistency of the time indicator was assessed to observe whether the differences in values are expected from one year to the next. In the case where there is an existence of larger difference, it suggests the need for further scrutiny. While large differences usually suggest some type of reporting error, it is also possible that the introduction of a new intervention might have contributed to a significant percentage increase in indicator values from one year to the next.

9- What do the authors mean by the indicator-pairs in and six-pairs of data items Table 2?

**Response:** Thank you for this valid question. Indicator-pairs in the Table 2 refer to a set of related data items or attributes that are expected to be consistent and maintain predictable relationship with each other. When assessing the quality of data, Authors examined indicator-pairs to identify any discrepancies that may exist within the two data items. By comparing the values of related data items, we can detect potential errors and inconsistencies. To analyze the consistency between related data, we enrolled six-pairs of data items and summarized if they exhibit the predicted relationship.

10- Please remove unnecessary tables.

**Response:** Thank you again for this valid suggestion. Unnecessary tables (Table 6 and 7 in the submitted manuscript) are transferred to supporting information section (named as S1 Table and S2 Table).

11- I think in the current study, the quality of health information has not been evaluated. If the authors did that, more information about data elements and quality indicators should be provided in the manuscript. Instead they assessed quality of facility reporting. Therefore, I suggest the authors to revise the title and the content of the manuscript to focus on quality of facility reporting not health data or information quality.

12- Apart from the statistical analysis, what are the innovative side of the research?

**Response:** Thank you for your suggestion. The authors made slight revisions to both the title and content of the manuscript, clearly stating that the review focuses on the quality of facility reporting. In the context of Nepal, there is limited evidence indicating that data quality assurance processes have been implemented for health facility data. At the sub-national level, specifically in Lumbini Province, multiple interventions for data quality assurance were carried out by both provincial and local authorities. However, the level of data quality remained unknown to the data generation and supervising authorities. Therefore, this assessment provides evidence to sub-national governments regarding the data quality status of health facilities. Additionally, this study aims to promote the practice of routine data quality reviews at the data generation level.

13- Please re-check the referencing style.

**Response:** Thank you for this valid comment. We re-checked and managed the referencing style as per the guideline.

**Responses to comments by Reviewer #2:** 
1- Authors did not thoroughly explain the research design deployed for their study. It is important for the scientific community to appreciate the research design deployed in order to draw appropriate links to the content of the study.

**Response:** Thank you for the suggestion. Authors now mentioned the research design for the study. In the methods section, the Authors also elaborated the basis for indicator selection and analysis processes.

2- The dimensions of data quality that the authors focused on were only completeness of the data, timeliness of the data and lastly the consistency/reliability of the data. However, using the same WHO data quality toolkit Module1 that the authors relied on, important dimension of data quality was conspicuously missing in the whole study. This dimension is the accuracy/validity of the data which is very important in unraveling whether the data faithfully reflects the actual level of service delivery conducted in the various health facilities included in the study. The manuscript should be modified to incorporate this important element in the assessment of quality data.

**Response:** We fully agree with the fact that we solely focused on the completeness and timeliness of MNCH datasets, as well as the consistency and reliability of key MNCH data items. We clearly mentioned in the study that it was based on a desk review of DHIS2 data. Due to the limitations of time and resources, it appears to be impossible to measure the accuracy of the data since it requires visits to health facilities and verification of data in multiple stages. This limitation was therefore mentioned in the discussion section of the manuscript.

3- The maternal indicators highlighted by the authors did not include HIV (with its attendant number of HIV positive mothers receiving ART), TB (with TB positive mothers receiving treatment) as well as mothers who tested positive for malaria either by microscopy or RDT) as these are very important maternal health indicators highlighted by the WHO data quality toolkit Module1. Once authors sourced their secondary data from the DHIS2, it is easier to retrieve the aforementioned variables.

**Response:** While we did refer to the WHO DQA module for selecting DQA indicators and assessing the consistency of reported data, this review was not entirely based on the WHO Data Quality Review (DQR) Standards. For example, we were unable to assess the timeliness and completeness of specific data items because DHIS2 does not have a function to measure this. Instead, we evaluated the completeness and timeliness of dataset-specific information as reflected in the DHIS2 platform. We selected the indicators take into account the MNCH continuum of care approach, their ability to cross-verify and measure data quality issues (as specified in data verification guide and DHIS2 data quality app), as well as their importance in program monitoring and evaluation, as stated in national and provincial policy documents.

4- Authors compared six pair of data in order to establish their relationship or association. The statistical basis of the comparison is defective as authors after their initial analysis as captured in table 9 ought to have deployed regression or other correlative statistical models to consolidate the association or otherwise observed.

**Response:** We analyzed six pairs of data to measure the consistency between related data. In order to review this, we defined the metrics according to the WHO's module on discrete desk review of data quality, and these metrics align with the data quality review practices utilized at the province and local levels in Lumbini province. Advanced statistical analysis was not employed in the assessment to ensure the manuscript remains understandable at the data generation level. The inability to utilize advanced analysis for this review is mentioned in the limitation section of the manuscript as well.

5- Authors must correct the spelling of Ethical as captured in their Ethical Considerations.

**Response:** Thank you. We corrected the spelling.

**Responses to comments by Reviewer #3:**

1- To reduce number of tables as it provides a distraction.

**Response:** Thank you, we reduced the number of tables as suggested.

2- Add more advance analysis such as t test or Anova to assess the temporal change in the related indicators to check how significant is the difference in the KPI.

**Response:** Thank you for your suggestion. We analyzed the three data quality dimensions (timeliness of facility reporting, consistency of facility reporting and the consistency of data). We relied on DHIS2 Pivot Table for the timeliness and completeness analysis, as this is the way that all the level of health system relies on defining HMIS reporting status. To review the consistency of data, we defined the metrics as per the WHO’s Module on Discrete desk review of data quality and these metrics are consistent with the data quality review practices employed by province and local levels in Lumbini province. More advance statistical analysis was not employed in the assessment to make the manuscript understandable up to health facility level. The unavailability to employ advanced analysis for this review are therefore mentioned in the limitation section of the manuscript as well.

3- Add more reference to the discussion part.

**Response:** Thank you for the feedback. We tried to review more studies and included references in the discussion section. Nevertheless, due to the limited availability of studies on routine MNCH data quality review, the authors may not be fully able to add more references to the pre-existing literature to demonstrate how the findings either align with or differ from previous research in some of the findings.

4- Were this study follows any guidelines such as STROBE? If no try to adapt it to assure the study is conducted in acceptable scientific writing.

**Response:** We followed the submission guideline developed by PLOS One journal. Referring to the Strengthening the Reporting of Observational studies in Epidemiology (STROBE) guideline also, we assure that the review is conducted in acceptable scientific writing.

**Responses to comments by Reviewer #4:**

1- The specific aim of the study was not clear. The methodology used to assessed the quality of reports did not meet the WHO Data Quality Review (DQR) Standards. Data quality assessment by WHO methodology is in two major phases
1. Desk Review
2.Site Assessment
By this article, it was only phase 1 that was carried out. I therefore suggest the author should at least sample the facilities for site assessment to authenticate the reports entered into DHIMS2 since the WHO data quality toolkit is the referenced tool used in the study

**Response:** We specified the aim of the study as a review of facility reported MNCH data. We conducted a desk review for this purpose and explicitly stated in the limitations section that we did not verify the paper based HMIS reports with DHIS2 data. While we did refer to the WHO DQA module for selecting DQA indicators and assessing the consistency of reported data, this review was not entirely based on the WHO Data Quality Review (DQR) Standards. For example, we were unable to assess the timeliness and completeness of specific data items because DHIS2 does not have a function to measure this. Instead, we evaluated the completeness and timeliness of dataset-specific information as reflected in the DHIS2 platform. Additionally, due to time and resource constraints, we did not conduct site visits, and these limitations are clearly discussed in the corresponding section.

**Additional Editor comments:**

Furthermore, the paper relies on routine data information from a routine data source of the Ministry of Health. As the data is collected through the routine health information management system of the government of Nepal. I request you provide a letter from the government (Ministry of Health) stating that they have provided consent for the publication of their information.

**Response:** As already mentioned in the manuscript, we received consent to access DHIS2 platform and publish the data from provincial Health Directorate under Ministry of Health, Lumbini Province. The Health Directorate in Lumbini Province is responsible for the overall information management in the province including DHIS2 user management. The authors also attached a letter from the government mentioning that they have provided consent for the publication of DHIS2 stored data.

Thank you so much!

The Authors
