# Supplementary material for: Quality of routine health facility data for monitoring maternal, newborn and child health indicators: A desk review of DHIS2 data in Lumbini Province, Nepal
Source: PLoS One. 2024 Apr 1;19(4):e0298101. doi: 10.1371/journal.pone.0298101 (PMC10984527; doi:10.1371/journal.pone.0298101)
Supplement: Attachment — Submitted filename: comment_LB.docx [file pone.0298101.s006.docx]

**Overall Suggestions:**

Although the authors have made considerable efforts to ensure the precision and rigor of this manuscript, I find myself not entirely satisfied with the methodology and analysis sections. Specifically, I would like to see more robust statistical analyses in the results section. For instance, incorporating powerful statistical tools like Bland-Altman Analysis for continuous numerical measurements in both datasets and employing the Kappa Statistic to measure agreement between the two systems (paper-based and digital) could strengthen the findings significantly. I kindly request the authors to enhance the analysis using these powerful statistical methods to achieve more robust and compelling results.

In line 17-19

DHIS2 is predominantly utilized by low and middle-income countries, making the statement more specific and targeted to its primary user base would be good.

Line 67-69

While your sentence effectively highlights specific data quality issues in information systems, I recommend a slight revision for clarity and conciseness. Consider rephrasing it as follows:

Specific data quality issues may arise, including incomplete, inconsistent, and irrelevant data, as well as imprecise estimates of the target population for coverage. These issues could limit the usefulness of the data for decision-makers.

Line 69-79

The authors pointed out that discrepancies between paper-based report findings and routine health information system findings. However, they did not provide any background information about the limitations of both systems and the existing gaps between them. I encourage them to explore and elaborate on the existing gaps between these two systems. This will significantly enhance the depth and credibility of their research, enabling readers to better understand the implications of the observed discrepancies.

Line 75-78

It is important to ensure proper referencing for the statement regarding Nepal's involvement in the mentioned initiatives, the development of action plans to reduce preventable deaths among mothers and children, and the considerable investment in strengthening information systems for performance management and service delivery. Adding appropriate references will not only provide credibility to the information but also give readers the opportunity to explore the sources and evidence supporting these claims.

Line 81-82

Is this claim made by the authors or by DHIS2? Proper referencing for this statement is necessary to clarify the source of the information and to ensure its accuracy and legitimacy.

Line 84-86

To prevent redundancy, kindly integrate this sentence with lines 82-84, for example: "The Ministry of Health and Population (MoHP) in Nepal introduced DHIS2 nationally as an electronic platform for Health Management Information System (HMIS) data management since 2016. Additionally, authors are encouraged to verify whether IHIMS and HMIS refer to the same system. If IHIMS represents recent changes to HMIS, please adjust the wording accordingly. Proper referencing for this information is also essential to ensure transparency and accuracy."

Line 105-107

Supporting reference for the statement required.

Line 105-111

I recommend that the authors incorporate these paragraphs into the introduction section. This is because the provided information pertains to provincial health indicators, which can be seamlessly linked with the section discussing the current gaps and the significance of the study in the introduction part. By doing so, the authors can create a cohesive and contextually relevant introduction, highlighting the importance of these indicators in framing the research objectives and addressing the study's significance.

Line 120

Kindly ensure uniformity throughout the entire manuscript by consistently using either "MCH" or "MNCH" terminology. The authors have used "MCH" in some sections and "MNCH" in others, creating inconsistency. It is essential to pick one term and apply it consistently across the entire document to maintain clarity and coherence

Line 121:

The authors mentioned that in 2021/22, DHIS2 contained a total of 12,509 reports. However, there is uncertainty regarding the definition of one report. Is it specified as one Maternal and Child Health (MCH) report submitted by one health facility on a monthly basis? To ensure clarity, the authors are requested to provide further clarification on the definition of a single report within DHIS2.

Line 125-126

Referencing(20) not required since its general statement.

Line 164-165

Authors explain completeness was approximately 100% but they didn’t mention what was the exact figure.It is recommended to mention exact figure in scientific studies.

Rather than displaying the dataset completeness results in a table, it is recommended for authors to present them using bar diagrams or other visual methods. This approach facilitates easier comparison of completeness levels across different datasets for readers.

Line 218

Please provide the full form of vaccines to make it easier to understand. For example, Penta 1 refers to the first dose of the Pentavalent Vaccine. Provide information on annex section.

Line 223

unit required for iron for e.g. Tablet/Capsule

Line 255-256

The authors have highlighted that the challenges faced in achieving completeness of DHIS2 reporting are not unique and have been documented in other studies as well. It is important to delve deeper into this issue to explore whether the difficulties are attributed to end users' discomfort with the system. Additionally, it is crucial to examine whether similar challenges exist in other countries, apart from Nepal.

Line 259-260

The authors asserted that the assessment result of timeliness is higher in this province compared to other provinces of Nepal; however, no specific reference data was provided in the entire manuscript. To support this claim, it is crucial to cite the relevant sources or present evidence within the manuscript to validate the statement.

Line 333-334

Please check the citation is well maintained as pr journal guideline.
