# Supplementary material for: Quality of routine health facility data for monitoring maternal, newborn and child health indicators: A desk review of DHIS2 data in Lumbini Province, Nepal
Source: PLoS One. 2024 Apr 1;19(4):e0298101. doi: 10.1371/journal.pone.0298101 (PMC10984527; doi:10.1371/journal.pone.0298101)
Supplement: Attachment — Submitted filename: PLOS ONE Article Review_PONE-D-2307291R1.docx [file pone.0298101.s007.docx]

**PLOS ONE Article Review**

**PONE-D-2307291R1**

Quality of Routine Health Facility Data for Monitoring Maternal, Newborn and Child Health Indicators: A Desk Review of DHIS2 Data in Lumbini Province, Nepal

**Reviewer’s comments**

**Summary and overall impression**

This article assesses the quality of routine health facility data for monitoring maternal, newborn and child health indicators in the Lumbini Province in Nepal using data from the District Health Information Software (DHIS2). The authors achieved this by adapting the WHO data quality toolkit Module1 to evaluate the completeness, timelines and consistency of data from the DHIS2 both at the province and district levels.

Key findings are that apart from consistency, the Lumbini province performed creditably on completeness and timeliness of data.

High quality data are fundamental for effective monitoring of important health indicators. Hence, the evaluation of quality of data is relevant. The manuscript however needs some improvements by addressing this and other comments accurately.

**Abstract**

***Conclusion****:*

1. Line 48 – Do not lead your readers wondering at this point. What specifically can be done to maintain consistency of data over time?

**Main Body**

***Introduction:***

2. Line 90 - consider inserting ……..reported data might *be* subjected……
3. Line 96 - I suggest *review* should be changed to *assess.*

***Study setting:***

4. Line 108 – why is *skilled birth attendant* in caps? Consider changing it to lower case.
5. Line 109 – change first letter of *incidence* to lower case.

***Study design and data sources:***

6. Line 124 – indicate the previous years you are referring to. This should be applicable to the entire document.

***Data analysis:***

7. Kindly expatiate on how data analysis was carried out.
8. Could authors provide the mean values for the preceding years?
9. The authors’ response to reviewer 1 comments 8 and 9 should be captured under the current heading (data analysis) for better clarity of how you proceeded with data analysis.
10. In addition to the authors’ explanation to the importance of the consistency of the mean values, you could also allude to the fact that consistency of the mean values is an indicator of reliability-meaning the greater probability that data source is trustworthy.

***Results:***

***Completeness of maternal, newborn and child health datasets &Timeliness of maternal, newborn and child health datasets:***

11. Since the report is disaggregated by district, (refer to tables 3 and 4) you should consider reporting the variations based on the districts. These variations could give an indication as to where the error is or source of data limitations within the province.
12. Provide plausible reasons for the variations observed.
13. Please indicate the sources of data for all your tables at the bottom of each table.
14. Make the province result distinct by separating it with a thick line or write the values in bold.

***Consistency over time:***

15. Move Lines 188 - 192 to the data analysis section.
16. Line 195-196 – the sentence should be preceded or end with *at the province level* to ensure a distinction between province and district level information.

***Accuracy of event reporting: outliers in the reference year:***

17. Line 210 – Authors should consider inserting values from the table to show your results. This is also applicable to the entire result section. Where necessary, insert some values from the result table as illustration so that your readers can follow you well.

***Discussion***:

18. Line 240 – The authors should consider changing *reviewed* to *assessed*
19. Line 255 – Authors should check the spelling if you are referring to the country in Africa called Rwanda and throughout the entire document.
20. Line 275 – there is an omission in the sentence, kindly check and revise accordingly.
21. Lines 280-282: At this point of your study, we expect the authors to be emphatic about what is happening in the province. It will be great and interesting to find this out and report it in your study findings.
22. Line 288 - …..*significant variations in these districts need further assessment*……..This could be a very good point for your recommendation section.
23. Lines 312-313: Do the authors consider these tests relevant for the study? If yes, why did they not perform the tests and if no why were they not done or why did the authors bring it up as a limitation? It is not appropriate to just indicate that you did not do it, provide justification for it.
24. Lines 315-317: If this not the gold standard, what is the gold standard and why was is not used in this study. Justify why you had to use the data regardless of the shortfalls (not meeting the gold standard)?
25. The authors may consider a sub-heading to capture your recommendations of this study.
