# Supplementary material for: Quality of routine health facility data for monitoring maternal, newborn and child health indicators: A desk review of DHIS2 data in Lumbini Province, Nepal
Source: PLoS One. 2024 Apr 1;19(4):e0298101. doi: 10.1371/journal.pone.0298101 (PMC10984527; doi:10.1371/journal.pone.0298101)
Supplement: Attachment — Submitted filename: Authors Response to Reviewers_Nov 2023.docx [file pone.0298101.s008.docx]

**Authors’ responses to review comments:**

At first, the authors are grateful to the editors and reviewers for taking their time and providing these interesting and constructive comments that help us improve the quality of our manuscript. With this, we have tried to address all the comments point-by-point in the revised manuscript as follows.

**Abstract**

***Conclusion****:*

1. Line 48 – Do not lead your readers wondering at this point. What specifically can be done to maintain consistency of data over time?

**Response:** Thank you for this question. Specific recommendations to maintain the consistency of data over time was mentioned in the revised manuscript as suggested.

**Main Body**

***Introduction:***

2. Line 90 - consider inserting ……..reported data might *be* subjected……
3. Line 96 - I suggest *review* should be changed to *assess.*

**Response:** Updated the manuscript as suggested.

***Study setting:***

4. Line 108 – why is *skilled birth attendant* in caps? Consider changing it to lower case.
5. Line 109 – change first letter of *incidence* to lower case.

**Response:** Revised as suggested

***Study design and data sources:***

6. Line 124 – indicate the previous years you are referring to. This should be applicable to the entire document.

**Response:** Indicated previous years: FY 2018/19 to 2020/21 clearly in the revised manuscript.

***Data analysis:***

7. Kindly expatiate on how data analysis was carried out.
8. Could authors provide the mean values for the preceding years?
9. The authors’ response to reviewer 1 comments 8 and 9 should be captured under the current heading (data analysis) for better clarity of how you proceeded with data analysis.
10. In addition to the authors’ explanation to the importance of the consistency of the mean values, you could also allude to the fact that consistency of the mean values is an indicator of reliability-meaning the greater probability that data source is trustworthy.

**Response:**

Metrics for data analysis of each domain are clearly mentioned in the Table 2 of manuscript. Analysis process employed are also mentioned in the narrative.

Mean values of preceding years are not separately mentioned in the manuscript, but the ratio of Year 4 to mean of Year 1-3 ((2021/22 to mean of 2018/19- 2020/21) was mentioned to analyze consistency over time. As per the comment, we would also like to mention the mean values of preceding years hereunder:

| **Organisation unit / data items** | **Mean values for the preceding years (2018/19- 2020/21)** | | | | | | | | |
| --- | --- | --- | --- | --- | --- | --- | --- | --- | --- |
|  | **4ANC as per protocol** | **Institutional delivery** | **Received incentive on transportation** | **4 PNC as per protocol** | **Children immunized with BCG** | **MR2 coverage** | **Diarrhoea cases treated with ORS and Zinc** | **New growth monitoring visit (0-11)** | **Exclusive breastfeeding** |
| RUKUM EAST | 561.67 | 616.0 | 581.3 | 416.0 | 1192.0 | 1127.0 | 1758.0 | 1610.0 | 550.0 |
| ROLPA | 3172.33 | 3479.3 | 3377.3 | 1973.0 | 5190.3 | 4821.0 | 6116.0 | 5532.3 | 3164.0 |
| PYUTHAN | 3189.33 | 3936.7 | 3936.3 | 1737.7 | 4959.3 | 4677.7 | 5280.0 | 6075.7 | 3592.0 |
| GULMI | 3486.67 | 2568.3 | 3214.7 | 1447.0 | 4269.3 | 4469.7 | 3560.3 | 6286.0 | 3032.7 |
| ARGHAKHANCHI | 2056.33 | 1377.3 | 1212.3 | 490.7 | 3105.0 | 3178.3 | 1638.0 | 4419.3 | 2204.0 |
| PALPA | 4415.67 | 5447.0 | 2700.3 | 1352.0 | 4127.0 | 4023.3 | 3212.7 | 4997.3 | 2719.7 |
| NAWALPARASI WEST | 4848.67 | 3189.0 | 2989.0 | 1155.3 | 5916.0 | 6364.0 | 3377.3 | 7618.7 | 6522.7 |
| RUPANDEHI | 16898.67 | 27621.3 | 17260.3 | 5877.3 | 26254.0 | 18448.3 | 7423.3 | 21447.0 | 9683.7 |
| KAPILBASTU | 7865.00 | 7477.3 | 6593.0 | 1870.3 | 13507.3 | 11614.7 | 11131.7 | 12312.3 | 7457.7 |
| DANG | 7666.00 | 8566.7 | 9492.0 | 2778.7 | 12425.0 | 11300.3 | 5509.0 | 10425.3 | 4356.0 |
| BANKE | 8506.00 | 19194.3 | 16671.3 | 3741.0 | 13391.3 | 10253.0 | 6826.0 | 9217.7 | 3719.7 |
| BARDIYA | 6181.33 | 5456.7 | 5205.0 | 3193.3 | 7911.7 | 7580.0 | 4385.3 | 8177.7 | 4072.0 |
| Lumbini Province | 68847.67 | 88930.0 | 73233.0 | 26032.3 | 102248.3 | 87857.3 | 60217.7 | 98119.3 | 51074.0 |

The authors’ response to reviewer 1 comments 8 and 9 are clearly mentioned under the data analysis heading in the revised manuscript.

Authors also included the recommended fact by the reviewer that the consistency of the mean values is an indicator of reliability, meaning a greater probability that the data source is trustworthy.

***Results:***

***Completeness of maternal, newborn and child health datasets &Timeliness of maternal, newborn and child health datasets:***

11. Since the report is disaggregated by district, (refer to tables 3 and 4) you should consider reporting the variations based on the districts. These variations could give an indication as to where the error is or source of data limitations within the province.
12. Provide plausible reasons for the variations observed.
13. Please indicate the sources of data for all your tables at the bottom of each table.
14. Make the province result distinct by separating it with a thick line or write the values in bold.

***Response:***

The authors accepted the comments and revised the manuscript as suggested. In the narrative of reporting completeness and timeliness, the variations between the districts are clearly mentioned. Additionally, plausible reasons for the variations are also included, specifically in the discussion section. The authors clearly indicated the sources of data for all figures and tables. Province results are written in bold to separate the values from district values.

***Consistency over time:***

15. Move Lines 188 - 192 to the data analysis section.
16. Line 195-196 – the sentence should be preceded or end with *at the province level* to ensure a distinction between province and district level information.

***Response:*** Addressed the comments

***Accuracy of event reporting: outliers in the reference year:***

17. Line 210 – Authors should consider inserting values from the table to show your results. This is also applicable to the entire result section. Where necessary, insert some values from the result table as illustration so that your readers can follow you well.

**Response:** Thank you for this valid comment. Authors now inserted the results from tables in the narrative as relevant.

***Discussion***:

18. Line 240 – The authors should consider changing *reviewed* to *assessed*

**Response:** Addressed

19. Line 255 – Authors should check the spelling if you are referring to the country in Africa called Rwanda and throughout the entire document.

**Response:** Checked and corrected

20. Line 275 – there is an omission in the sentence, kindly check and revise accordingly.

**Response:** Checked and corrected

21. Lines 280-282: At this point of your study, we expect the authors to be emphatic about what is happening in the province. It will be great and interesting to find this out and report it in your study findings.

**Response:** Thank you for this valid comment. Authors mentioned the programmatic implication of the introduction of the PNC home visit program in not maintaining consistency over time for the data item - PNC visits as per protocol.

22. Line 288 - …..*significant variations in these districts need further assessment*……..This could be a very good point for your recommendation section.

**Response:** This point has been added in the recommendation section.

23. Lines 312-313: Do the authors consider these tests relevant for the study? If yes, why did they not perform the tests and if no why were they not done or why did the authors bring it up as a limitation? It is not appropriate to just indicate that you did not do it, provide justification for it.

**Response:** Authors do not actually consider these tests compulsory for such a review. The authors added this limitation based on suggestions from the first reviewers. In fact, the authors want this document to remain understandable at the data generation level (i.e., health facility and first-level data managers), where complex statistical tests are less relevant. The reason is now clearly mentioned in the manuscript.

24. Lines 315-317: If this not the gold standard, what is the gold standard and why was is not used in this study. Justify why you had to use the data regardless of the shortfalls (not meeting the gold standard)?

**Response:** Authors wanted to make it clear that two sources of data were compared to provide relevant references for assessing the external consistency of routine data, even though DHS is a household sample survey and DHIS2 reported data are facility-reported data. For the comparison, there could also be space to compare the DHIS2 data with other facility-based survey data. However, in the case of Nepal, no such surveys were conducted, allowing for the comparison of MNCH-related indicators.

Reviewer #5: Although the authors have made considerable efforts to ensure the precision and rigor of this manuscript, I find myself not entirely satisfied with the methodology and analysis sections. Specifically, I would like to see more robust statistical analyses in the results section. For instance, incorporating powerful statistical tools like Bland-Altman Analysis for continuous numerical measurements in both datasets and employing the Kappa Statistic to measure agreement between the two systems (paper-based and digital) could strengthen the findings significantly. I kindly request the authors to enhance the analysis using these powerful statistical methods to achieve more robust and compelling results.

**Response:** As suggested by the reviewer, the analysis section has been made more comprehensive by including measurement metrics and additional information in the analysis tables. The authors aimed to simplify the analysis to ensure understanding at the data generation level (i.e., health facility and first-level data managers), where complex statistical tests are less relevant. The study's objective was specified as a review of facility-reported MNCH data. To achieve this, a desk review was conducted. It is explicitly stated in the limitations section that the paper-based HMIS reports were not verified with DHIS2 data.

In line 17-19

DHIS2 is predominantly utilized by low and middle-income countries, making the statement more specific and targeted to its primary user base would be good.

**Response:** Ay accepting the comment, the authors revised the manuscript.

Line 67-69

While your sentence effectively highlights specific data quality issues in information systems, I recommend a slight revision for clarity and conciseness. Consider rephrasing it as follows:

Specific data quality issues may arise, including incomplete, inconsistent, and irrelevant data, as well as imprecise estimates of the target population for coverage. These issues could limit the usefulness of the data for decision-makers.

**Response:** This comment is well taken and revised as suggested.

Line 69-79

The authors pointed out that discrepancies between paper-based report findings and routine health information system findings. However, they did not provide any background information about the limitations of both systems and the existing gaps between them. I encourage them to explore and elaborate on the existing gaps between these two systems. This will significantly enhance the depth and credibility of their research, enabling readers to better understand the implications of the observed discrepancies.

**Response:** Authors mentioned about the observed discrepancies between the coverage estimated derived from routine information systems and population based surveys. To further elaborate, Authors discussed more in discussion section of the manuscript.

Line 75-78

It is important to ensure proper referencing for the statement regarding Nepal's involvement in the mentioned initiatives, the development of action plans to reduce preventable deaths among mothers and children, and the considerable investment in strengthening information systems for performance management and service delivery. Adding appropriate references will not only provide credibility to the information but also give readers the opportunity to explore the sources and evidence supporting these claims.

**Response:** Authors made proper referencing for the statement regarding Nepal’s involvement in initiatives to reduce preventable deaths among mothers and children, as well as related investments in strengthening information systems.

Line 81-82

Is this claim made by the authors or by DHIS2? Proper referencing for this statement is necessary to clarify the source of the information and to ensure its accuracy and legitimacy.

**Response:** This is the claim based on a literature review. The authors supported this statement, ensuring accuracy and legitimacy through proper citation.

Line 84-86

To prevent redundancy, kindly integrate this sentence with lines 82-84, for example: "The Ministry of Health and Population (MoHP) in Nepal introduced DHIS2 nationally as an electronic platform for Health Management Information System (HMIS) data management since 2016. Additionally, authors are encouraged to verify whether IHIMS and HMIS refer to the same system. If IHIMS represents recent changes to HMIS, please adjust the wording accordingly. Proper referencing for this information is also essential to ensure transparency and accuracy."

**Response:** This comment is well taken and addressed as suggested.

Line 105-107

Supporting reference for the statement required.

**Response:** Reference provided for the status of specific SDG indicators.

Line 105-111

I recommend that the authors incorporate these paragraphs into the introduction section. This is because the provided information pertains to provincial health indicators, which can be seamlessly linked with the section discussing the current gaps and the significance of the study in the introduction part. By doing so, the authors can create a cohesive and contextually relevant introduction, highlighting the importance of these indicators in framing the research objectives and addressing the study's significance.

**Response:** Incorporated the lines 105-111 in introduction section as suggested.

Line 120

Kindly ensure uniformity throughout the entire manuscript by consistently using either "MCH" or "MNCH" terminology. The authors have used "MCH" in some sections and "MNCH" in others, creating inconsistency. It is essential to pick one term and apply it consistently across the entire document to maintain clarity and coherence

**Response:** Comment addressed.

Line 121:

The authors mentioned that in 2021/22, DHIS2 contained a total of 12,509 reports. However, there is uncertainty regarding the definition of one report. Is it specified as one Maternal and Child Health (MCH) report submitted by one health facility on a monthly basis? To ensure clarity, the authors are requested to provide further clarification on the definition of a single report within DHIS2.

**Response:** Report in the manuscript refers to the monthly report submitted by each health facility (HMIS 9.3/9.4/9.5). This report includes individual sections for immunization, safe motherhood, nutrition, and IMNCI.

Line 125-126

Referencing (20) not required since its general statement.

**Response:** Removed the reference as suggested.

Line 164-165

Authors explain completeness was approximately 100% but they didn’t mention what was the exact figure.It is recommended to mention exact figure in scientific studies.

**Response:** Authors addressed the comment by mentioning exact figures.

Rather than displaying the dataset completeness results in a table, it is recommended for authors to present them using bar diagrams or other visual methods. This approach facilitates easier comparison of completeness levels across different datasets for readers.

**Response:** Authors presented the completeness and timeliness related data in bar diagram as recommended.

Line 218

Please provide the full form of vaccines to make it easier to understand. For example, Penta 1 refers to the first dose of the Pentavalent Vaccine. Provide information on annex section.

**Response:** Addressed the comment

Line 223

unit required for iron for e.g. Tablet/Capsule

**Response:** Addressed the comment

Line 255-256

The authors have highlighted that the challenges faced in achieving completeness of DHIS2 reporting are not unique and have been documented in other studies as well. It is important to delve deeper into this issue to explore whether the difficulties are attributed to end users' discomfort with the system. Additionally, it is crucial to examine whether similar challenges exist in other countries, apart from Nepal.

**Response:** By accepting the suggestion, the authors explored the presence of similar challenges in other countries and cited them in the manuscript. However, this study has a limitation: conducting an in-depth analysis of challenges related to the completeness of data in Nepal based on a review of DHIS2 data would not be relevant.

Line 259-260

The authors asserted that the assessment result of timeliness is higher in this province compared to other provinces of Nepal; however, no specific reference data was provided in the entire manuscript. To support this claim, it is crucial to cite the relevant sources or present evidence within the manuscript to validate the statement.

**Response:** Yes, result of timeliness is higher in Lumbini province compared to other provinces of Nepal as reflected in HMIS online platform (DHIS2). To support this statement, the Authors now provided the reference.

Line 333-334

Please check the citation is well maintained as pr journal guideline.

**Response:** Corrected, thank you

**Reviewer #6:** MORE DESCRIPTION OF THE MANUFACTURING PROGRAM WILL ADD INFORMATION TO THE READER FOR PEOPLE IN OTHER COUNTRIES.

**Response:** By accepting the comment, authors tried to add additional information about the program as relevant.

**Reviewer #7:** the author has adjusted the manuscript according to the suggestions of the previous reviewer's input and corrected the manuscript. well done

**Reviewer #8:** (No Response)

**Reviewer #9**
1. Abstract:
a) Methods section: My suggestion is to not label the study as cross-sectional. The study is ecological in nature since monthly aggregated data is utilized and there is no access to individual data. Another option is to use the term “descriptive study”.
b) Methods section: In a sentence, please mention the number of data elements selected for each objective. Readers might first assume that all 23 data items were used for all objectives, which isn’t the case.

**Response:**

a) Revised the study as descriptive.
b) Mentioned the number of data elements selected for each objective as suggested.

2. Main Text:
a) Methods: Same comment as 1a regarding study design.
b) Line 138: Please add a comma before “and Safe Motherhood”. Otherwise, “Nutrition and Safe Motherhood” is interpreted as a single dataset.
c) For all tables, please add footnotes explaining the abbreviations used in them. Readers will find it cumbersome to search for the abbreviations in the main text. For example, in Table 1 please explain ANC, PNC, what is the meaning of ‘x’ in the column etc. Also, mention the relevant years for each table.
d) Table 2: While assessing outliers the authors have selected ±2SD. This method is apt for normally distributed data. No tests of normality or graphical visualizations were conducted and selecting ±2SD as the benchmark could be problematic. It would be better to conduct some basic tests to verify normality which isn’t complex. Additionally, please mention if selecting ±33% and ±10% as the benchmark for other metrics of consistency is specifically mentioned in the WHO data quality report card (or any other reference). If not, wjhat was the reasoning behind the criteria?
e) In the first paragraph of the results section (lines 183-184), it is mentioned that “5 out of 12 districts have 100% completeness for nutrition and IMNCI datasets (in each) and 4 out of 10 districts have 100% completeness for safe motherhood datasets”. However, in Table 3, I count 7 provinces with 100% completion for nutrition and IMNCI individually. Similarly, the results for safe motherhood do not match. Table 3 shows 8 out of 12 districts with 100% completion (and not 4 out of 10). If the total districts for safe motherhood were 10, identify the dropped districts in the table/text and provide a brief explanation as to the reason for exclusion. Please correct/verify the results.
f) While reporting timeliness the authors mention “By district, 9 out of 12 districts have more than 90% reporting rate on time for the selected datasets.” By my calculations, all districts, except Rukum East, have an average above 90%, i.e. 11 out of 12 have more than a 90% score. Please correct/verify.
g) Table 6: It wasn’t clear to me how the results reported in the final row were arrived at after calculating the total outliers in every district. Provide a brief footnote with an explanation.
h) Line 331: The sentence mentions “no one of the coverage rates are flagged” instead of “none”.

**Response:** Thank you for the in-depth review and comments. Authors have addressed all the comments in the revised manuscript and summary of responses are also mentioned hereunder:

a) Addressed the comment as suggested
b) Added comma before “and Safe Motherhood”.
c) Mentioned the footnotes mentioning the meaning of symbols used, as well as explained the abbreviations where needed.
d) Extreme or moderate outliers (i.e. moderate outliers if values are ± 2–3 SD from the mean or > 3.5 on modified z-score method) are identified based on the criteria set by WHO Data Quality Assurance: Framework and Metrics 2022. As suggested by reviewer, we also performed normality test that showed that the data followed symmetrical distribution and this process is now clearly mentioned in the revised manuscript. Reference for selecting ±33% and ±10% as the benchmark for other metrics of consistency is mentioned in the WHO Data Quality Assurance: Framework and Metrics 2022 and also cited in the manuscript (reference number 21).
e) Thank you for pointing the mistake. We now corrected the error with careful observation of data.
f) Verified the data and made a minor update in the narrative, written as “By district, 9 out of 12 districts have more than a 90% reporting rate on time for each of the selected datasets.
g) Footnote with explanation of calculating Average % of moderate outliers was clearly mentioned in the revised manuscript.
h) Corrected as suggested.

Thank you
