# Supplementary material for: Quality of routine health facility data for monitoring maternal, newborn and child health indicators: A desk review of DHIS2 data in Lumbini Province, Nepal
Source: PLoS One. 2024 Apr 1;19(4):e0298101. doi: 10.1371/journal.pone.0298101 (PMC10984527; doi:10.1371/journal.pone.0298101)
Supplement: Attachment — Submitted filename: Reviewer comments.docx [file pone.0298101.s004.docx]

The paper is well written with interesting topic.

The last sample size and national coverage is outstanding. However, some comments might improve this manuscript.

The comment are as follows:

1- To reduce number of tables as it provides a distraction.
2- Add more advance analysis such as t test or Anova to assess the temporal change in the related indicators to check how significant is the difference in the KPI.
3- Add more reference to the discussion part.
4- Were this study follows any guidelines such as STROBE? If no try to adapt it to assure the study is conducted in acceptable scientific writing.
